# Ion Sputter Induced Interfacial Reaction in Prototypical Metal-GaN System

**DOI:** 10.1038/s41598-018-26734-5

**Published:** 2018-06-04

**Authors:** Rong Huang, Fangsen Li, Tong Liu, Yanfei Zhao, Yafeng Zhu, Yang Shen, Xiaoming Lu, Zengli Huang, Jianping Liu, Liqun Zhang, Shuming Zhang, Zhanping Li, An Dingsun, Hui Yang

**Affiliations:** 1 Vacuum Interconnected Nanotech Workstation (Nano-X), Suzhou Institute of Nano-Tech and Nano-Bionics (SINANO), Chinese Academy of Sciences (CAS), Suzhou, 215123 China; 2 Key Laboratory of Nanodevices and Applications, Chinese Academy of Sciences (CAS), Suzhou, 215123 China; 3 Analysis Center, Tsinghua University, Beijing, 100084 China

## Abstract

Contact property is now becoming to be a key factor for achieving high performance and high reliability in GaN-based III-V semiconductor devices. Energetic ion sputter, as an effective interface probe, is widely used to profile the metal/GaN contacts for interfacial analysis and process optimization. However, the details of ion-induced interfacial reaction, as well as the formation of sputter by-products at the interfaces are still unclear. Here by combining state-of-the-art Ar^+^ ion sputter with *in-situ* X-ray photoelectron spectroscopy (XPS) and *ex-situ* high resolution transmission electron microscopy (HRTEM), we have observed clearly not only the ion-induced chemical state changes at interface, but also the by-products at the prototypical Ti/GaN system. For the first time, we identified the formation of a metallic Ga layer at the GaO_x_/GaN interface. At the Ti/GaO_x_ interface, TiC_x_ components were also detected due to the reaction between metal Ti and surface-adsorbed C species. Our study reveals that the corresponding core level binding energy and peak intensity obtained from ion sputter depth profile should be treated with much caution, since they will be changed due to ion-induced interface reactions and formation of by-products during ion bombardment.

## Introduction

Ion-sputter depth profiling is currently a ubiquitous technique applied in various areas, and widely used to resolve layered information of heterostructure^1,2^, such as metal/GaN and Al_x_Ga_1-x_N/GaN. Many efforts were made to understand changes resulted from high energetic ion incident, such as preferential sputtering, atomic mixing in collisional cascade, interfacial reactions and segregation. Studies on the metal contact properties in GaN-based III-V semiconductor devices^3–11^ are of particular importance not only for fundamental researches, but also for process developments. To get very low Ohmic contact resistance between metals and GaN is a prerequisite for devices to achieve high performance and high reliability. X-ray photoelectron spectroscopy (XPS) depth profiles have indicated inter-diffusion between metal layers and GaN^3,4,12–15^, such as Ga out-diffusion and Au in-diffusion, which were often considered as the root causes for device failures. Core level (CL) shift or Fermi level shift^3,4,13–17^ that happened at metal/GaN interface, which was often referred to explain the alignment of band structure in the contact junction, may be a complex situation after considering what we found here.

There have been extensive researches on ion-sputter depth profiles of metal/GaN systems to explore the contact properties^3–13^, however, interfacial reactions induced by ion-sputter were seldom studied in depth and details. Here, we applied *in-situ* XPS to analyze the ion sputtered surfaces after removal of each layer step by step. As surface roughness is one of the physically well-defined factors to induce distortions of the original in-depth distribution^1^, we designed a contact structure on purpose with a thin metal layer (i.e. 10 nm Ti layer here) with a thin cap layer (i.e. 10 nm Pd layer here), rather than the common Ohmic contact structure with thick metal layers^8,9^. Benefit from this special design, the roughness at interface is nearly the same as the initial surface, promising us credible XPS results during the ion sputter. GaO_x_ layer were always observed on GaN surface after exposure to air. More interestingly, we found that ion sputter induced the formation of metallic Ga layer and TiC_x_ compounds at the metal/GaN interface. These ion-sputter-induced interfacial reactions and products alter the chemical states and compositions on GaN surface, which could be characterized by the corresponding core level peak intensity and binding energy. This phenomenon must be considered with caution either for data analysis of depth profiling or properly understanding of the contact properties.

## Results and Discussion

**Figure 1:**
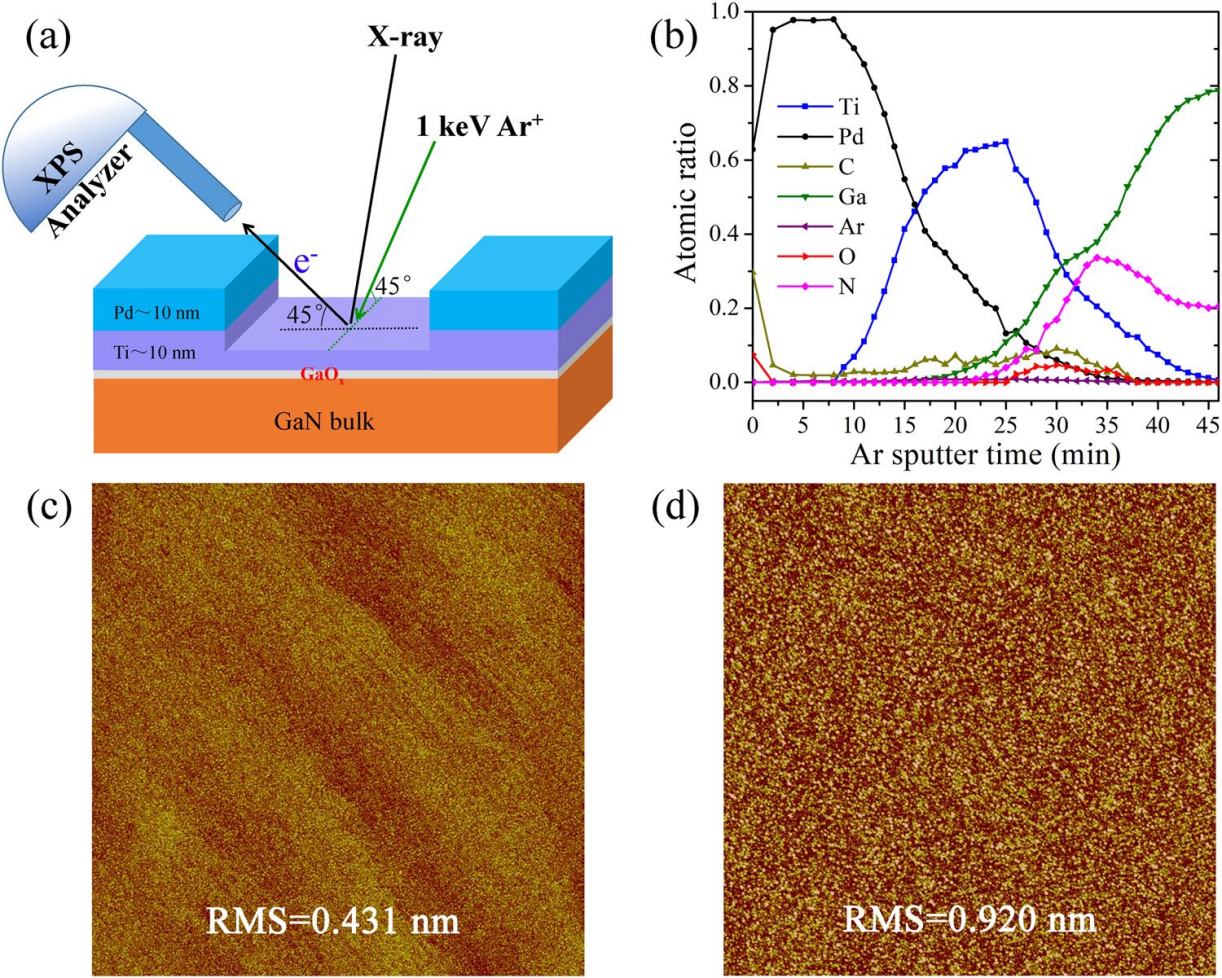
Ar^+^ ion sputter and *in-situ* XPS depth profile on sample N2. (**a**) Schematic drawing of sputtering geometry. (**b**) XPS depth profile with incident 1 keV Ar^+^. The surface morphology (5 × 5 μm^2^) imaged by AFM after (**c**) 30 min and (**d**) 45 min ion sputtering, respectively.

First of all, we focus on Ar^+^ ion sputter experiment on Pd/Ti/GaN sample with ICP treatment (Labeled as N2). Schematic diagram in Fig. 1a shows the configuration of ion sputtering and XPS measurement process. Both Ar^+^ beam incident angle and photoelectron detection angle are 45° relative to the normal direction, with incident X-ray and analyzed photoelectron on one geometric plane and incident Ar^+^ ion on the plane perpendicular to it. A crater of ~ 3 × 3 mm^2^ was created by the sputtered Ar^+^ ion. The sputter rate mainly depends on the properties of sample materials, the kinetic energy and current of Ar^+^ ions. With fixed scan area and incident angle, in this experiment, sputter time is used to refer the crater depth for relatively comparing similar samples sputtered with identical conditions. To explore the reactions at interfaces of metal/GaN, ion sputter and analysis on the samples were carried out alternately with an interval of 2 min sputter at the beginning and then 1 min follow behind when approaching to the interfaces. Surface morphologies of sample N2 with different Ar^+^ ion (1 keV) sputter time were shown in Fig. 1(c,d). After 30 min sputter (near the Ti/GaN interface), the root mean square (RMS) roughness is only 0.43 nm, which is almost the same as the original ICP etched surface (RMS ~ 0.440 nm). Therefore, we could ignore possible XPS peak broadening and depth resolution deviation induced by the surface roughness at the Ti/GaN interface. After another 15 min sputter, the RMS slightly increased to 0.92 nm when sputtering across Ti/GaN interface and into the GaN bulk, which probably because of the formation of TiC_x_ compounds (would be demonstrated below) of high hardness at the interface. For the purpose of comparison, we also profiled a typical Ohmic contact structure with thick metal layers of 50 nm Ti / 50 nm Pt/100 nm Au on GaN surfaces. The crater roughness at the Ti/GaN interface is ~ 4 nm as measured by *ex-situ* AFM (see Fig. S1a in Supplementary Information), indicating that deeper sputter leads to larger roughness and poorer depth resolution. To illustrate detailed interfacial reaction, therefore, thin metal layers of Pd (10 nm)/Ti (10 nm) were deposited on *n*-GaN by a UHV magnetron sputter process. With this simulative structure, the main intrinsic phenomena that we are more interested in at the Ti/GaN interface should be similar to the one formed with thicker metal layers.

Figure 1b shows the processed XPS depth profile of sample N2, where Pd 3d, Ti 2p, Ga 3d, Ar 2p, C 1 s, O 1 s and N 1 s peaks are used for determining the contents of Pd, Ti, Ga, Ar, C, O, and N in the films, respectively. The atomic ratio of each component was calculated based on areas of these peaks and their corresponding relative sensitivity factors (RSF). Since O 1 s peak overlaps with Pd 3p3/2 peak, the area of O 1 s was deduced by subtracting Pd 3p3/2 portion from total area, which was calculated from Pd 3d peak and its RSF instead. With improvement of the XPS depth resolution, three dominant layers can be clearly resolved. The atomic ratio of O is quite low in the metal layers, even less than 1% in Ti layer. On the contrary, oxygen content could be higher than 50% in Ti layer if not have a Pd protection layer capped (see Fig. S1d in Supplementary Information), indicating that 10 nm Pd layer is good enough to protect Ti thin film from oxidation. At the Ti/GaN interface in Fig. 1b, oxygen content increased to about 6% due to the existence of GaO_x_ on GaN surface before metal deposition, which was also confirmed by subsequent HRTEM and TOF-SIMS results. On the other hand, surface-adsorbed C species were almost completely sputtered away at the first two minutes, dropping to a very low level (~2%) in the metal layers, and then slightly increased at Ti/GaN interface.

**Figure 2:**
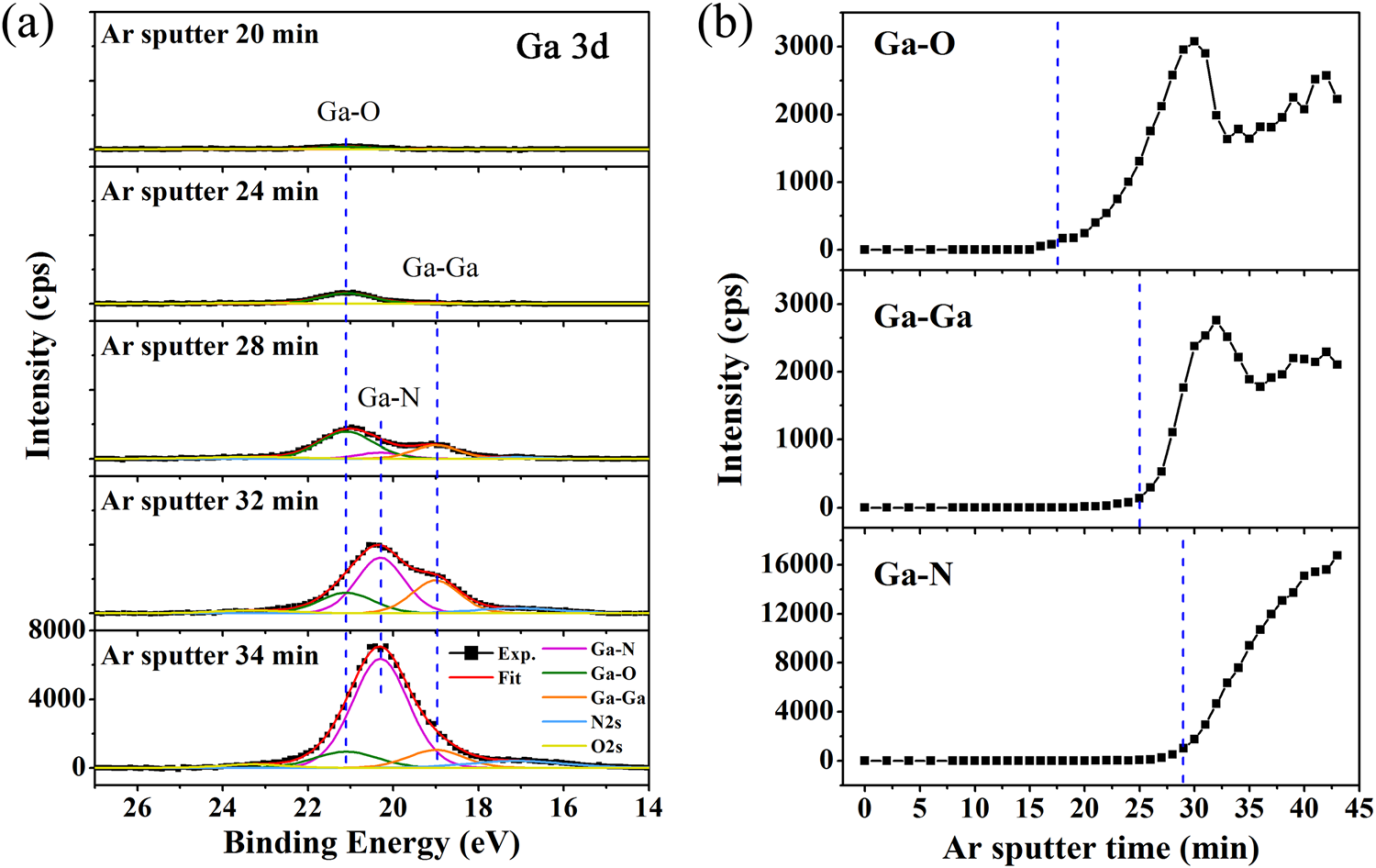
Ion depth profile of Ga element. (**a**) A serial of Ga 3d core level spectra during sputtering with binding energy in the range of 14–27 eV. After subtracting the Shirley background, the Ga 3d peak could be de-convoluted into five peaks. (**b**) Depth profile of different Ga components (Ga-O, Ga-Ga and Ga-N) calcuated from Ga 3d peak as function of sputter time.

XPS measurement, with characteristic photoelectron peaks correspond to the core level electron configuration of atoms in the materials, is also well known as the technique for Chemical State Analysis^18^. It is often used to detect chemical state changes in the surface layer of sample that might be processed by different methods, such as Ar^+^ ion sputter in this work. We extracted Ga 3d signal from the depth profile and drew it in Fig. 2a, where intensity of Ga 3d increases with sputter time apparently, and also the peak position shifts during sputtering. Considering the fact that advanced dual-beam neutralization was used to compensate charging effect and to stabilize the sample surface potential, the peak shift should be caused mainly by the change of chemical states. A typical Ga 3d spectrum (low panel in Fig. 2a) is de-convoluted into five peaks: Ga-O bond at 21.1 eV, Ga-N bond at 20.3 eV, Ga-Ga bond at 19.0 eV, N 2 s at 17.1 eV, and O 2 s at about 23.3 eV (see detail in Supplementary Information Fig. S2). The principle of de-convolution is shown in the inset of Fig. S2, which was applied to all the Ga 3d spectra detected during sputtering.

At the beginning, as shown in Fig. 2a, Ga 3d peak is contributed only from Ga-O bonds^19^ on the GaN surface. In fact, this kind of Ga-polar GaN surface was always wetted with Ga-Ga metal layer after MOCVD growth, and then oxidized quickly into GaO_x_ when exposed to air^20,21^. In evolution of Ga 3d peak, the appearance of Ga-O bond prior to Ti-Ga metal bond suggests limited out-diffusion of Ga atoms, which are usually observed after annealing^3,4,12–15^. More interestingly, as the sputtering went on, we noticed a clear shoulder or an additional component appearing at lower binding energy side of the Ga 3d peak, located around 19.0 ± 0.2 eV. This additional component rose before the emergence of Ga-N bond from GaN bulk, which should be attributed to metallic Ga-Ga bonds. In Fig. 2b, we have plotted the intensity of different Ga components as a function of sputter time. The Ga-O intensity reaches its maximum at sputter time of 30 min, about 2.5 min ahead of Ga-Ga intensity, suggesting that metallic Ga mainly exists at the GaO_x_/GaN interface, where N-Ga-O bonds may be mixed and then broken by the ion bombardment^22^. In previous XPS depth profile, metallic Ga-Ga signals were also observed at Ga_2_O_3_ (Gd_2_O_3_)-GaN^5^ and Ti (Au)-GaN systems^6^, but there has no explanation so far about how and from where the metal Ga atoms generated at interfaces. On bare GaN surfaces, previous experimental studies^10,23,24^ and molecular dynamic simulations^25,26^ have shown that ion bombardment can directly induce a Ga-rich surface or a metallic Ga layer due to Ga-N bond breaking. Even metallic Ga droplets with a height of ~15 nm could form after bombarding with 1 keV Ar^+^ ions, which was attributed to Ga atoms released from Ga-N bonds breaking, as reported in literature^24^.

**Figure 3:**
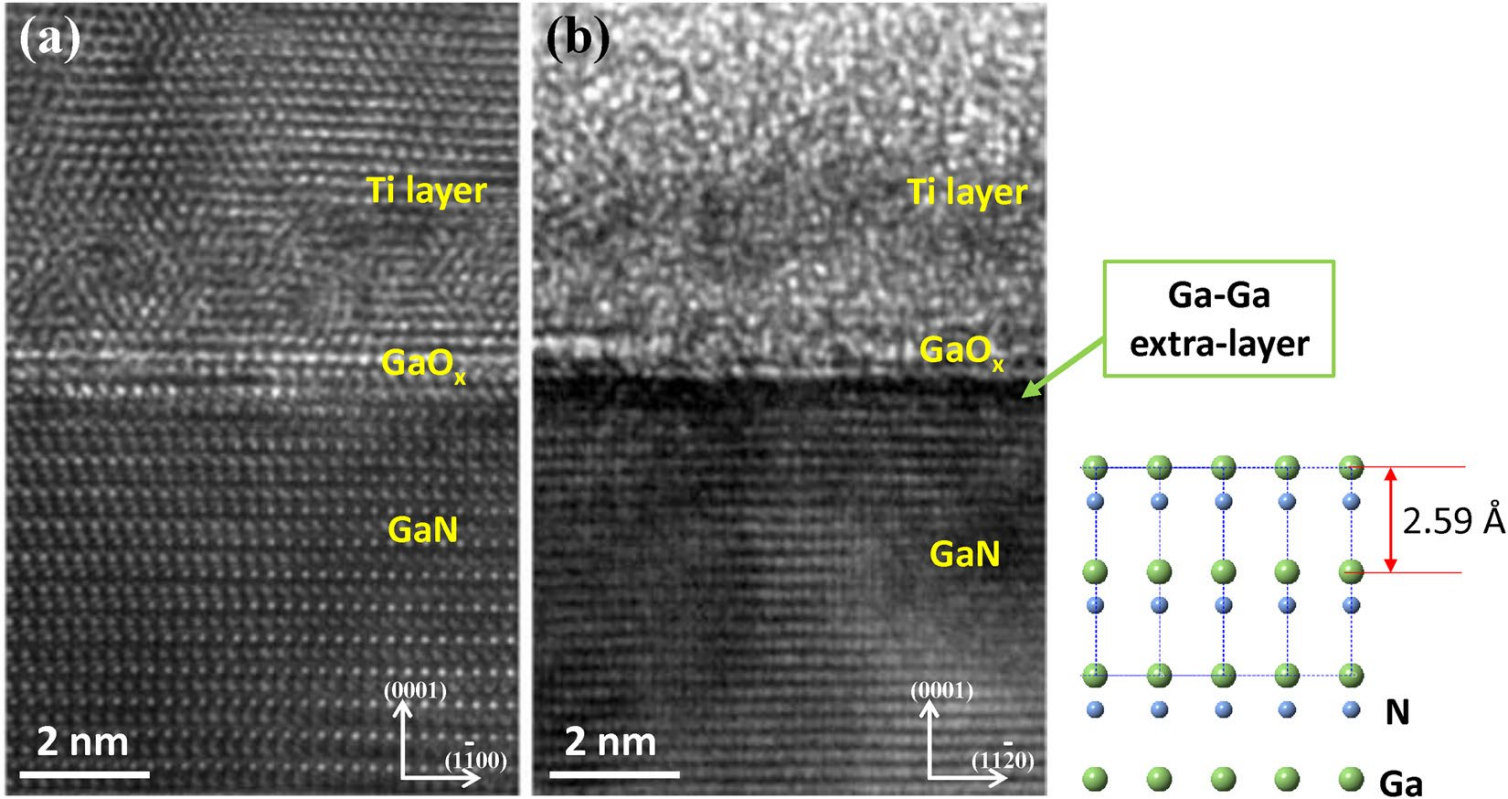
The HRTEM observation of Ti-GaN interface, before (**a**) and after (**b**) Ar^+^ ion sputter on sample N2. Inset: GaN (0001) ball model.

To further confirm and explain the formation of metallic Ga extra layer below GaO_x_ layer, we carried out *ex-situ* high resolution transmission electron microscopy (HRTEM) studies. Figure 3a shows the HRTEM image of Ti/GaN interface without Ar^+^ ion sputter. The as-deposited Ti layer exhibits polycrystalline morphology with small crystalline grains. Between Ti layer and GaN, a thin GaO_x_ layer with thickness of ~1 nm was clearly observed with enlarged lattice constant *c* ~2.7 Å, which is consistent with previous TEM results^7,27^. For the ion sputtered sample, Fig. 3b shows the HRTEM image of Ti-GaN interface after 30 min Ar sputter on sample N2, where Ga-O signal reached maximum and the center of the 3 × 3 mm^2^ sputtered crater was picked up to prepare the HRTEM sample. We failed to resolve atomic crystalline structure in the Ti metal layer, because the structure of Ti layer was disordered by the incident Ar^+^ ions and cascade collision, as well as further oxidation of Ti metal during the sample preparation. The meaningful thing is a darker layer observed clearly below the GaO_x_ layer in Fig. 3b, comparing with the no-sputter area in Fig. 3a. This was identified as metallic Ga layer, in consistence with our XPS observations.

Ar^+^ sputter depth profile has been widely used to study the contact properties at metal/GaN interfaces. For the first time, ion bombardment induced Ga-Ga layer formation at GaO_x_/GaN interface was observed in this work. The thickness of the metallic Ga layer is only ~0.5 nm, suggesting a mild ion-induced reaction at the interface. It should be pointed out that this phenomenon that happened in metal/GaN contact layer is not only limited to Ti, but also true for other metals, such as Au/GaN contacts^6^. The formation of Ga metallic by-product during ion sputtering process strongly suggests us to be careful not only in characterization of metal/GaN contact properties, such as core level peak shift and contact potential barrier^3,4,13–17^, but also in design of device processes with ion sputter or etching involved.

**Figure 4:**
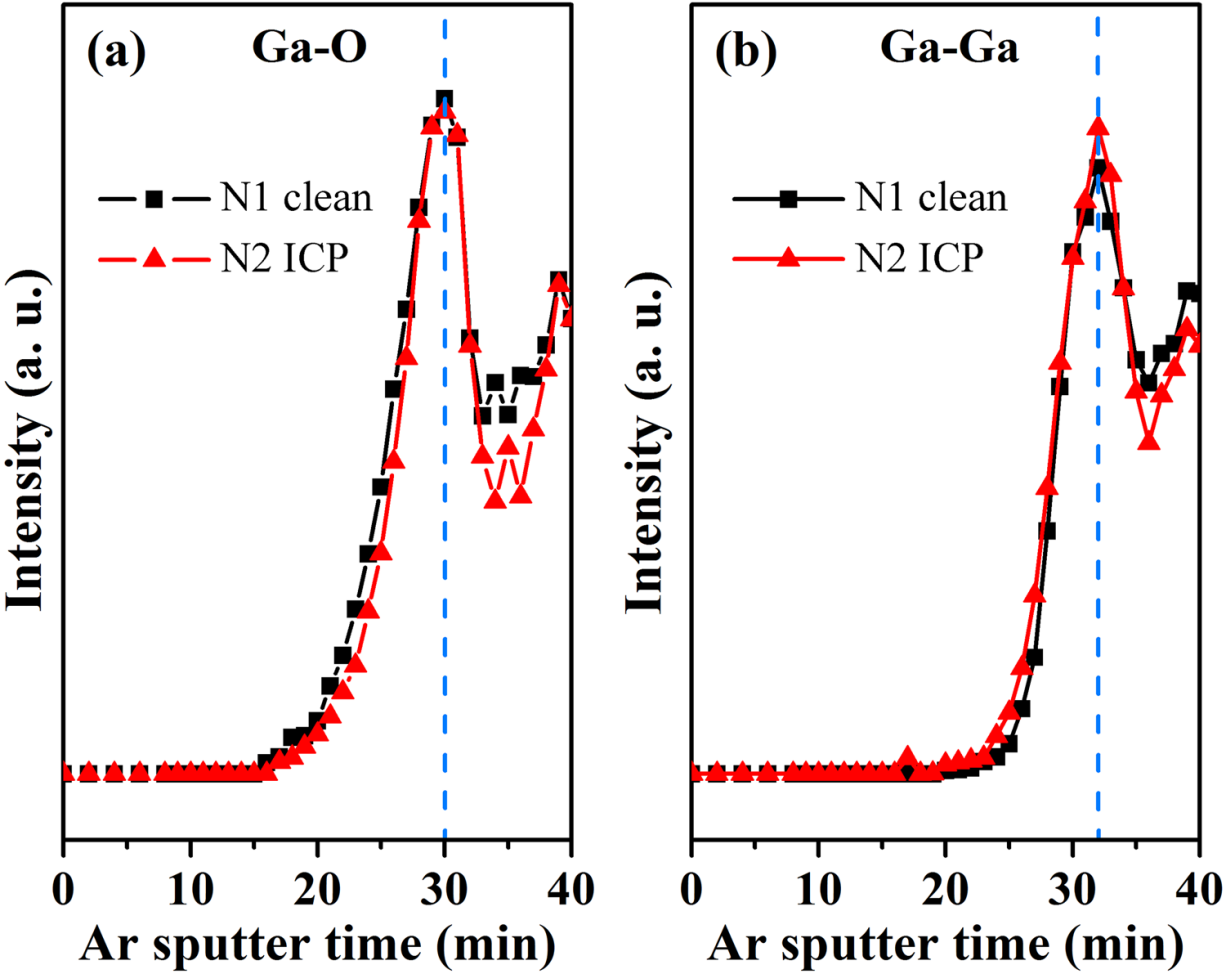
The depth profile of (**a**) Ga-O and (**b**) Ga-Ga component in Ga 3d core level spectra on N1, N2 samples.

For comparison, depth profiles for sample N1 and sample N2 were overlapped together in Fig. 4. It was found that GaO_x_ layer always exists on GaN surface regardless of how it was treated. In both samples, Ga-O signal was observed nearly at the same time (Fig. 4a), and Ga-Ga signal exhibits similar curve as Ga-O component, but peaks of Ga-Ga intensity exist at a little deeper place (Fig. 4b). It is worth to note that not only the intensity but also the area of metallic Ga layer is nearly the same for samples N1 and N2. Similar as that reported by Wang, L. *et al*. in ref.^27^, our TOF-SIMS analysis results show that the thickness of GaO_x_ layer on GaN surface was nearly doubled after ICP treatment, (see Fig. S3 in Supplementary Information). XPS depth profile with Ar^+^ ion sputter here may not have enough depth resolution to distinguish GaO_x_ layers with slight difference in thickness on sample surfaces of N1 and N2. Nevertheless, we are still able to conclude that Ga-Ga layer emerged at GaO_x_/GaN interface by combined XPS and HRTEM results.

**Figure 5:**
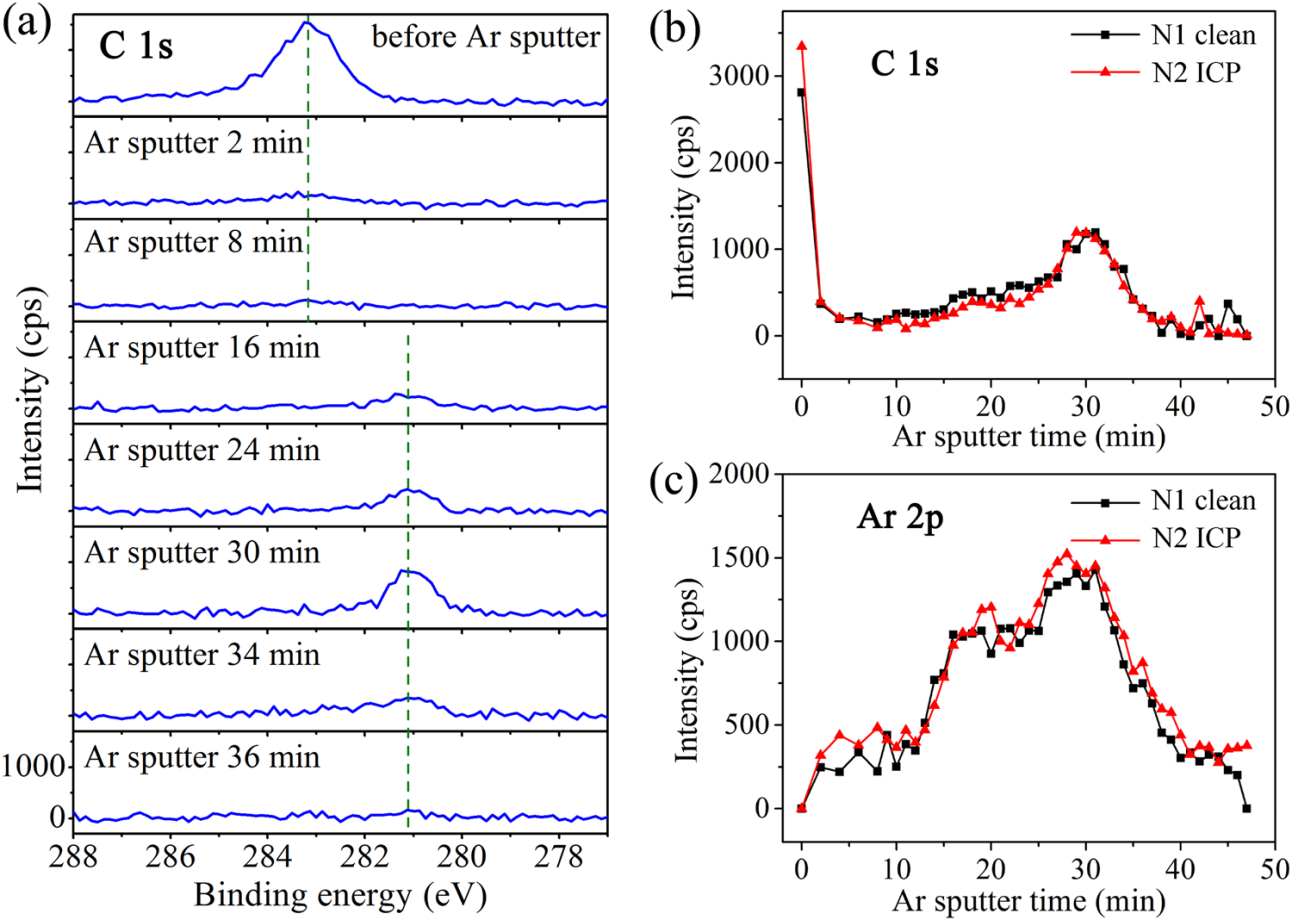
Depth profile of C and Ar components. (**a**) C 1 s core level spectra during sputtering with binding energy in the range of 277 eV–288 eV. (**b**) Depth profile of C 1 s component. (**c**) Depth profile of Ar 2p component.

Besides metallic Ga component, the C 1 s peak changes too in both intensity and peak position following to Ar^+^ ion sputter process, as shown in Fig. 5. Strong C 1 s signal was observed on as-loaded sample surfaces, due to the adsorption of carbon-containing compound from ambient. C components were sputtered away quickly and only detectable as trace in the metal layers. Regarding to the very low signal to noise ratio of C 1 s peak, the binding energy (BE) of other core-level peaks are hard to be calibrated with respect to the C-C bond (BE = 284.8 eV). Instead, we relied on the dual-beam neutralizing system to compensate potential charging effect on the sample surface that may cause additional peak shifts. The C 1 s peak enhanced again when approaching to the Ti/GaN interface, probably because of C adsorption on the GaN surface before metal deposition. However, the C 1 s peak shifts obviously from 284.8 eV to a lower binding energy of ~281.1 eV, which is regarded to the formation of TiC_x_ (~281.7 eV in ref.^28^) at the interface. The energetic Ar^+^ might break the C-H or C-C bonds of carbon-containing compounds, and then promote metallic Ti to form TiC_x_. Samples N1 and N2 exhibited similar C depth profiles with intensity maximum at ~30 min of sputter. This is in accordance with the depth profile of Ga-O in Fig. 4, and further proofing the Ar^+^ induced interfacial reaction of TiC_x_ at the Ti/GaN interface. The formation of TiC_x_ may also affect the reliability of Ti/GaN contact characterizations.

Similarly, we paid attention to Ar distribution in the depth profile of Pd/Ti/GaN structure and distinct Ar implant on different layers were observed. Figure 5b shows the Ar 2p profile with maximal intensity at the Ti/GaN interface, consisting with the fact of ion-induced reactions happened at the Ti/GaN interface during Ar^+^ sputtering.

## Conclusion

In conclusion, we studied in details the effects of energetic ion sputter on metal/GaN contacts, especially the ion-induced interface reactions and by-product formation. By combining state-of-art Ar^+^ ion sputter with surface sensitive XPS and *ex-situ* HRTEM characterization, we observed that ion bombardment on sample surface not only causes physical collisions, such as confusion of Ti crystalline structure and implant of Ar atoms, but also breaks some chemical bonds, such as N-Ga-O and C-H/C-C. Bonds breaking by energetic ions is one of the keys for formation of metallic and carbonate components at the metal/GaN interfaces. In further, we identified the formation of metallic Ga layer at GaO_x_/GaN interface and TiC_x_ components at Ti/GaO_x_ interface, regardless of how the GaN surfaces were treated before metal deposition. The ion-induced interfacial reaction and by-product formation during ion sputter should be carefully considered not only for depth profiling and data analysis of metal/GaN contacts, but also for the design and optimization of device processes that have energetic ions involved, such as plasma/ion-beam etchings.

## Methods

### Sample preparation

The Ga-polar (0001) GaN wafer was grown by metal-organic chemical vapor deposition (MOCVD) onto a *c*-plane (0001) sapphire substrate with a 2 μm Si-doped *n*-GaN epitaxial layer on top of a 2 μm undoped GaN buffer layer. The procedure of GaN wafer growth has been described elsewhere^29^. We adopted two different types of surface treatment methods (Labeled as N1, N2) on GaN prior to metal deposition. Both GaN samples were firstly cleaned by acetone and then isopropanol for several times, after then rinsed thoroughly in DI water and blew dry with pure N_2_ gas. Subsequently, they (N1 and N2) were cleaned in acid solution (HCl: H_2_O_2_: H_2_O = 1:1:5) for 20 min and then followed by alkaline solution (48% KOH, 80 °C) for 15 min. Thereafter, N2 samples were further treated by inductively coupled plasma (ICP) for 30 s with 18 sccm Cl_2_, 1000 W ICP power, and 50 W RF power, which was optimized to form good Ohmic contact between GaN and metal Ti^30^. The uniform metal layers of Ti and Pd were sequentially deposited on N1 and N2 samples in an ultra-high vacuum (UHV) magnetron sputter chamber with a base pressure of 5 × 10^−10^ Torr. The deposition rate is 0.66 Å/s and 1.08 Å/s, respectively.

### Ion sputter and XPS characterization

Combining Ar^+^ sputter and a surface analytical technique alternatively to get sample information layer by layer is known as ion sputter depth profile^1^. The Pd/Ti/GaN sample was profiled by sputtering obliquely with nominated 1 keV kinetic energy under a partial pressure of ~5.5 × 10^−5^ Torr, and then probed *in-situ* by XPS. XPS measurements were carried out in an ultra-high vacuum (UHV) chamber at a pressure of 3 × 10^−9^ Torr or better, equipped with a hemispherical electron energy analyzer (PHI 5000, VersaProbe, ULVAC-PHI) and a monochromatic Al K_α_ X-ray source of 1486.6 eV. The Ar^+^ sputter gun and dual-beam charging compensation system are also attached to the UHV chamber, focusing on the XPS analysis area of sample. The sputtered area is about 3 × 3 mm^2^, and XPS analytical area is about 0.1 × 0.1 mm^2^. The high-resolution XPS spectra were collected with a pass energy of 58.7 eV, and an energy step size of 0.125 eV. To eliminate charge effect induced peak shift, we applied the dual-beam neutralization technique for sample compensation during the data collection. After subtracting a Shirley-type background, the spectra are curve-fitted with a combination of Gaussian (70%) and Lorentzian (30%) line shapes. The surface morphology after ion bombardment was characterized *ex-situ* by atomic force microscopy (AFM). Time-of-Flight Secondary Ion Mass Spectrometry (TOF-SIMS) analyses were carried out by using an IONTOF apparatus (TOF.SIMS 5–100), equipped with a Bi liquid metal ion source as the primary beam gun. For high depth resolution, Bi_3_^++^ ion beam with an energy of 30 keV and a current of 4.5 nA was used here. The 500 eV Cs^+^ ion source with a beam current of 5 nA was used as the sputter gun to physically remove the material layer by layer.

### HRTEM characterization

Interface atomic structures were observed by using an *ex-situ* high resolution transmission electron microscopy (HRTEM) on a Talos (FEI) setup, with 200 keV voltage and at room temperature. Samples for the HRTEM characterizations were prepared at the locations of outside (without Ar sputter) and inside the center of Ar sputtered crater (~3 × 3 mm^2^) by using focused ion beam (FIB) method. Cross-sectional lamella was thinned down to 100 nm by a focused Ga^+^ beam at an accelerating voltage of 30 kV and a current decreasing from maximum of 1 nA to minimum of 0.1 nA, followed by fine polishing at an accelerating voltage of 2 kV and a small current of 8.6 pA. All HRTEM images were corrected for drift with the lattice constant of *c* ∼ 5.178 Å in the bulk.

## Electronic supplementary material

Supplementary Information

## References

[1] Hofmann S (2014). Sputter depth profiling: past, present, and future. Surface and Interface Analysis.

[2] Lisowski W (2014). XPS method as a useful tool for studies of quantum well epitaxial materials: Chemical composition and thermal stability of InGaN/GaN multilayers. Journal of Alloys and Compounds.

[3] Kim SK, Han JC, Seong T-Y (2016). Thermally stable Ti/Al-based ohmic contacts to N-polar n-GaN by using an indium interlayer. Japanese Journal of Applied Physics.

[4] Park JS, Han J, Seong TY (2015). Formation of low resistance Ti/Al-based ohmic contacts on (11-22) semipolar n-type GaN. Journal of Alloys and Compounds.

[5] Lay TS (2005). Depth-profile study of the electronic structures at Ga_2_O_3_(Gd_2_O_3_) and Gd_2_O_3_–GaN interfaces by X-ray photoelectron spectroscopy. Journal of Crystal Growth.

[6] Dumonta J, Monroy E, Muñoz E, Caudano R, Sporken R (2001). Investigation of metal–GaNand metal–AlGaN contacts by XPS depth profiles and by electrical measurements. Journal of Crystal Growth.

[7] Ishikawa H (1997). Effects of surface treatments and metal work functions on electrical properties at p-GaN/metal interfaces. Journal of Applied Physics.

[8] Chen J, Brewer WD (2015). Ohmic Contacts on p-GaN. Advanced Electronic Materials.

[9] Giuseppe G, Ferdinando I, Fabrizio R (2016). Ohmic contacts to Gallium Nitride materials. Applied Surface Science.

[10] Lai YH (2001). Sputtering and etching of GaN surfaces. Journal of Physical Chemistry B.

[11] Sun X (2015). High spectral response of self-driven GaN-based detectors by controlling the contact barrier height. Scientific Reports.

[12] kkaya A, Esmer L, Kantar BB, Cetin H, Ayyildiz E (2014). Effect of thermal annealing on electrical and structural properties of Ni/Au/n-GaN Schottky contacts. Microelectronic Engineering.

[13] Han SC (2013). Ohmic Contacts to N-Face p-GaN Using Ni/Au for the Fabrication of Polarization Inverted Light-Emitting Diodes. Journal of Nanoscience and Nanotechnology.

[14] Reddy NNK, Reddy VR, Choi CJ (2011). Influence of rapid thermal annealing effect on electrical and structural properties of Pd/Ru Schottky contacts to n-type GaN. Materials Chemistry and Physics.

[15] Reddy VR, Ravinandan M, Koteswara Rao P, Choi C-J (2008). Effects of thermal annealing on the electrical and structural properties of Pt/Mo Schottky contacts on n-type GaN. Journal of Materials Science: Materials in Electronics.

[16] Janga J-S, Seong T-Y, Jeon S-R (2007). Formation mechanisms of low-resistance and thermally stable Pd/Ni/Pd/Ru Ohmic contacts to Mg-doped Al_0.15_Ga_0.85_N. Applied Physics Letters.

[17] Song JO, Kwak JS, Seong TY (2006). Improvement of the ohmic characteristics of Pd contacts to p-type GaN using an Ag interlayer. Semiconductor Science and Technology.

[18] Fadley CS (2010). X-ray photoelectron spectroscopy: Progress and perspectives. Journal of Electron Spectroscopy and Related Phenomena.

[19] Gangopadhyay S (2014). Surface oxidation of GaN(0001): Nitrogen plasma-assisted cleaning for ultrahigh vacuum applications. Journal of Vacuum Science & Technology A: Vacuum, Surfaces, and Films.

[20] Hattori AN, Endo K, Hattori K, Daimon H (2010). Surface treatments toward obtaining clean GaN(0001) from commercial hydride vapor phase epitaxy and metal-organic chemical vapor deposition substrates in ultrahigh vacuum. Applied Surface Science.

[21] Ma J (1996). Photoemission spectroscopy studies of the surface of GaN films grown by vapor phase epitaxy. Applied Physics Letters.

[22] Luo, Y.-R. *Comprehensive Handbook of Chemical Bond Energies*. (CRC Press, 2007).

[23] Deenapanray PNK, Petravić M, Kim KJ, Kim B, Li G (2003). Compositional changes on GaN surfaces under low-energy ion bombardment studied by synchrotron-based spectroscopies. Applied Physics Letters.

[24] Venugopal V, Upadhyaya K, Kumar K, Shivaprasad SM (2014). Ion induced compositional changes and nanodroplet formation on GaN surface. Applied Surface Science.

[25] Despiau-Pujo E, Chabert P (2010). MD simulations of GaN sputtering by Ar^+^ ions: Ion-induced damage and near-surface modification under continuous bombardment. Journal of Vacuum Science & Technology A.

[26] Emilie Despiau-Pujo PC (2010). Low energy Ar^+^ bombardment of GaN surfaces: A statistical study of ion reflection and sputtering. Journal of Vacuum Science & Technology A.

[27] Wang, L. *et al*. Reduction of the resistivity of Ag/p-GaN contact by progressive breakdown of the interfacial contamination layer. *Journal of Applied Physics* **118** (2015).

[28] Galuska AA, Uht JC, Marquez N (1988). Reactive and Nonreactive Ion Mixing of Ti Films on Carbon Substrates. Journal of Vacuum Science & Technology a-Vacuum Surfaces and Films.

[29] Zhou K (2015). Influence of substrate surface defects on the homoepitaxial growth of GaN (0001) by metalorganic vapor phase epitaxy. Journal of Crystal Growth.

[30] Huang R (2018). Angular dependent XPS study of surface band bending on Ga-polar n-GaN. Applied Surface Science.

