# Supplementary material for: Ion Sputter Induced Interfacial Reaction in Prototypical Metal-GaN System
Source: Sci Rep. 2018 Jun 4;8:8521. doi: 10.1038/s41598-018-26734-5 (PMC5986764; doi:10.1038/s41598-018-26734-5)
Supplement: Supplementary file 1 — Supplementary Information [file 41598_2018_26734_MOESM1_ESM.pdf]

Supplementary Information for:

Ion Sputter Induced Interfacial Reaction in Prototypical Metal-GaN System

Rong Huang[1], Fangsen Li[1,*], Tong Liu[1], Yanfei Zhao[1], Yafeng Zhu[1], Yang Shen[1], Xiaoming Lu[1], Zengli Huang[1], Jianping Liu[2], Liqun Zhang[2], Shuming Zhang[2], Zhanping Li[3], An Dingsun[1,*], Hui Yang[1, 2]

[1] Vacuum Interconnected Nanotech Workstation (Nano-X), Suzhou Institute of Nano-Tech and Nano-Bionics (SINANO), Chinese Academy of Sciences (CAS), Suzhou 215123, China

[2] Key Laboratory of Nanodevices and Applications, Chinese Academy of Sciences (CAS), Suzhou 215123, China

[3] Analysis Center, Tsinghua University, Beijing 100084, China

*E-mail: adingsun2014@sinano.ac.cn; fsli2015@sinano.ac.cn

The supplementary information includes:

1) Figs. S1 to S4

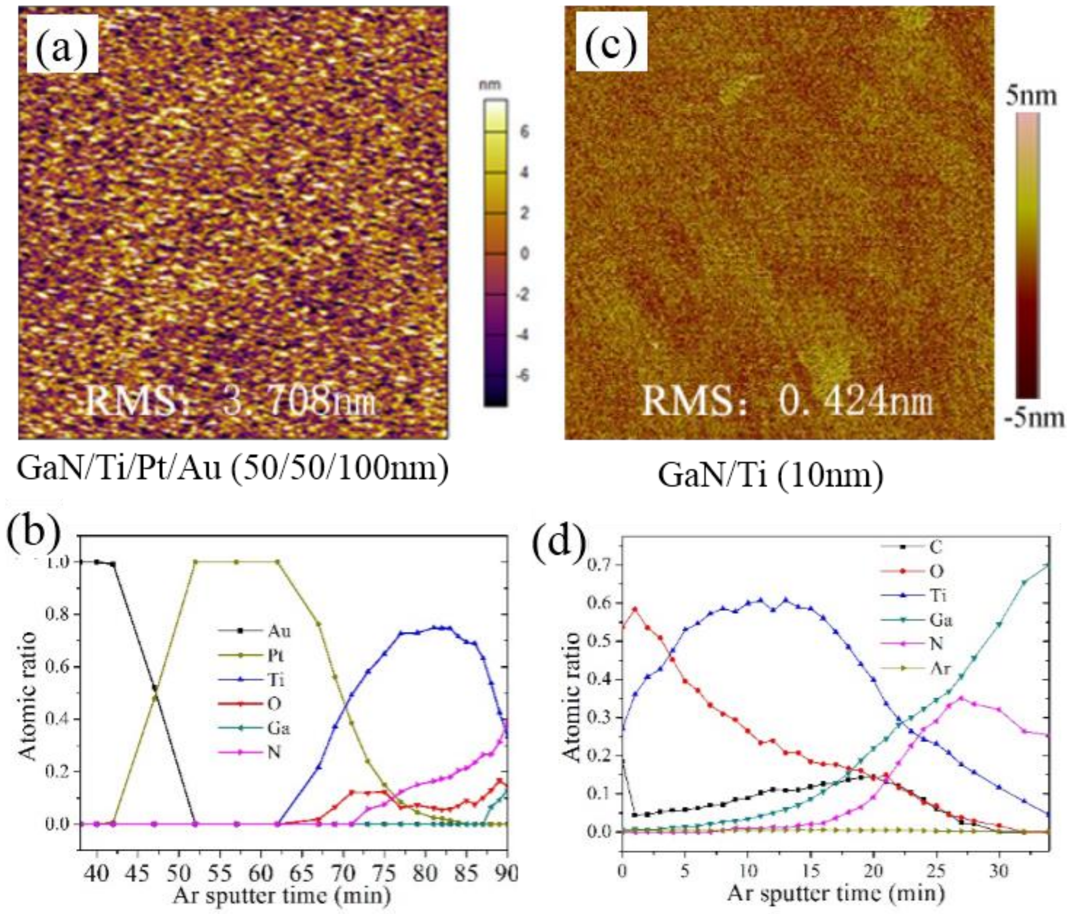

Fig. S1 Surface morphology (5×5 μm^2) imaged by AFM at Ti/GaN interface, from (a) normal Ohmic contact structure of GaN/50 nm Ti/ 50 nm Pt/100 nm Au, and (c) GaN/ 10 nm Ti. (b, d) XPS depth profile of these two structures, respectively.

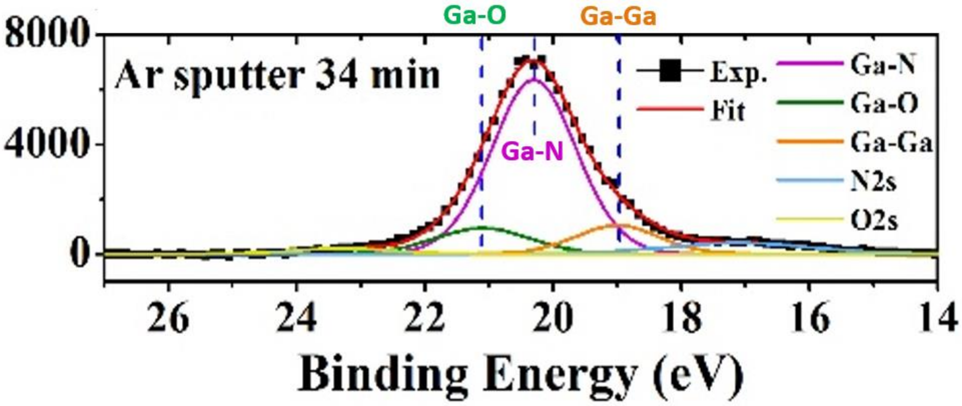

The principle of de-convolution

Ga 3d	Ga-N (A)	Ga-O (B)	Ga-Ga (C)	N 2s (D)
Peak/eV	A	A+0.8	A-1.6	A-3.2
FWHM/eV	A	A+0.2	A*1	D

Fig. S2 Detailed Ga 3d peak after 34 min sputter and the applied de-convolution principle.

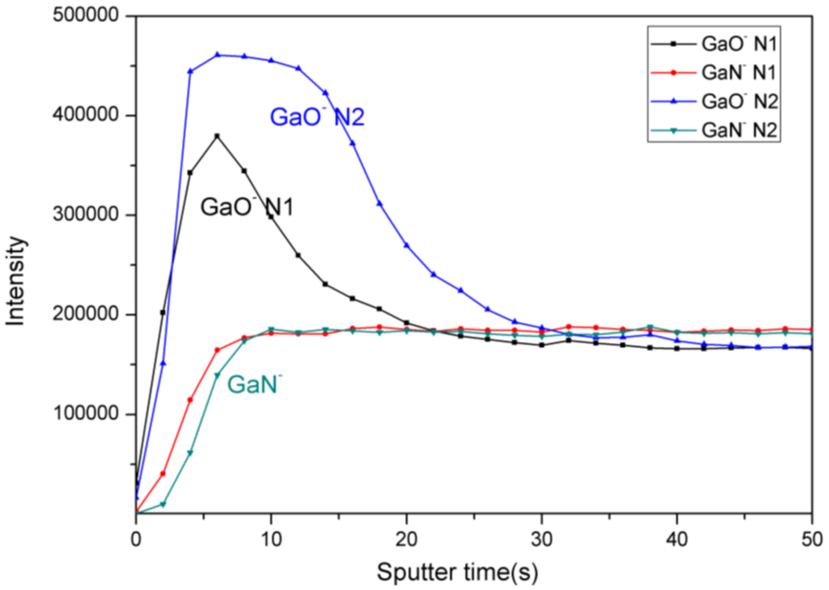

Fig. S3 TOF-SIMS experiment showing GaO$^-$ signal from bare GaN surface after different surface treatment (N1 and N2). The thickness of GaO layer nearly doubled in N2 sample.

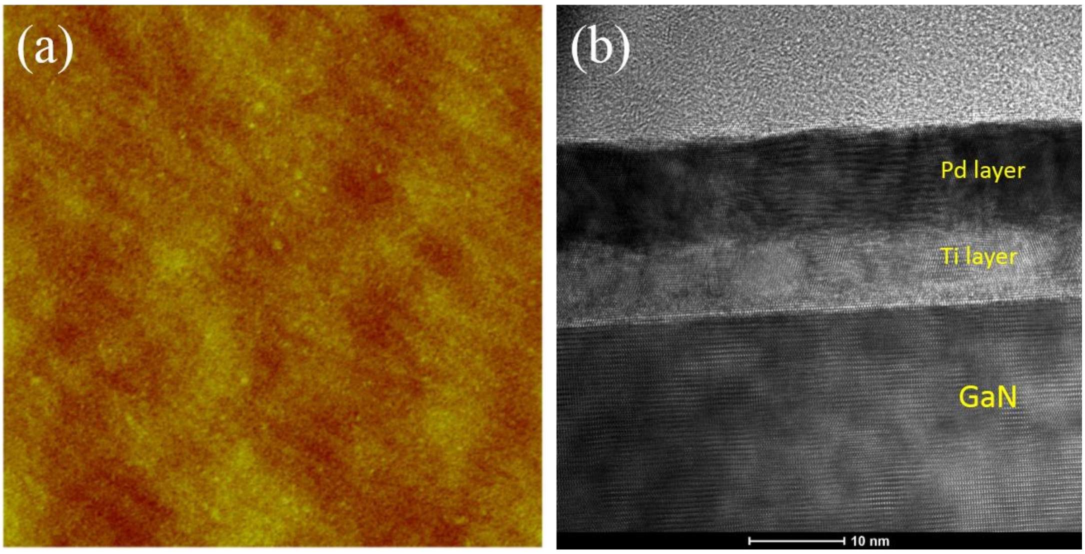

Fig. S4 (a) The surface morphology of deposited thin Ti film (~3×3 μm^2, RMS~0.544 nm). (b) Large-scale TEM observation of cross section of Pd/Ti/GaN, showing uniform metal layers on GaN surface.
